# Perinatal Intracranial Hemorrhage as a Rare Presentation of Plasminogen Activator Inhibitor-1 (PAI-1) Deficiency: A Case Report

**DOI:** 10.7759/cureus.80888

**Published:** 2025-03-20

**Authors:** Naini Puri, Aayushi Joshi, Shantanu Shubham, Syed Moiz Ahmed, Richa Joshi, Ankur Kapoor, Divya Mishra, Girish Gupta

**Affiliations:** 1 Pediatrics, Graphic Era Institute of Medical Sciences, Dehradun, IND; 2 Neonatology, Graphic Era Institute of Medical Sciences, Dehradun, IND; 3 Neurosurgery, Graphic Era Institute of Medical Sciences, Dehradun, IND; 4 Obstetrics and Gynaecology, Graphic Era Institute of Medical Sciences, Dehradun, IND

**Keywords:** neonatal intracranial hemorrhage, neonatal stroke, perinatal stroke, plasminogen activator inhibitor 1, ventricular dilatation

## Abstract

Perinatal stroke is a major cause of neonatal neurological impairment, but spontaneous intracranial hemorrhage due to plasminogen activator inhibitor-1 (PAI-1) deficiency is rare. We report a 28-day-old term female neonate who presented with seizures, irritability, and altered sensorium, later diagnosed with severe intraventricular and intracerebral hemorrhage (ICH). Extensive investigations ruled out common etiologies, leading to genetic testing that identified a heterozygous *SERPINE1* gene variant, confirming PAI-1 deficiency. Management included mechanical ventilation, external ventricular drainage, anticonvulsants, and tranexamic acid. Despite intensive care, the neonate developed cystic encephalomalacia and motor deficits. This case emphasizes on the importance of considering PAI-1 deficiency in unexplained neonatal hemorrhage. Genetic diagnosis and antifibrinolytic therapy may improve outcomes, though long-term neurodevelopmental impairment remains a concern. Multidisciplinary rehabilitation, parental counseling, and structured follow-up are crucial, and further research is needed to define optimal management strategies.

## Introduction

Perinatal stroke is a localized vascular injury to the brain that occurs from the fetal stage through the neonatal period, specifically between the 20th week of gestation and the first 28 days of postnatal life [1,2]. The incidence of perinatal stroke is approximately 1 in 1,000 live births, with significant morbidity, as it is the leading cause of hemiparetic cerebral palsy [3]. Perinatal stroke predominantly affects term infants, whereas preterm neonates are more susceptible to distinct brain injuries associated with developmental immaturity and hemodynamic instability. Unlike other neonatal vascular conditions, perinatal stroke does not encompass hypoxic-ischemic encephalopathy (HIE), which results from widespread hypoperfusion, nor does it include extra-axial hemorrhages such as subarachnoid, subdural, or epidural hemorrhages [4]. Intracranial hemorrhage is a major contributor to poor neurological outcomes in infants, as it disrupts brain development during a critical phase of structural and functional maturation [5,6].

The classification of perinatal stroke is determined by the timing of the injury (either fetal or neonatal), the underlying mechanism (ischemic or hemorrhagic), and the onset of clinical symptoms (immediate or delayed). It is broadly categorized into arterial ischemic, hemorrhagic, or venous stroke, with further classification based on the timing of presentation [7,8]. Neonatal arterial ischemic stroke (NAIS), neonatal hemorrhagic stroke (NHS), and neonatal cerebral sinus venous thrombosis (NCSVT) typically manifest early in the neonatal period, often presenting with symptoms such as seizures and encephalopathy. In contrast, the remaining three types of perinatal stroke usually become apparent after the first month of life and are primarily identified through neuroimaging findings. These include arterial presumed perinatal ischemic stroke (APPIS), presumed perinatal hemorrhagic stroke (PPHS), and periventricular venous infarction (PVI), which is considered a fetal stroke. Delayed-onset cases often present with hemiparesis as a primary symptom [9].

PAI-1 deficiency is a rare congenital disorder of fibrinolysis characterized by defective inhibition of tissue plasminogen activator (tPA) and urokinase plasminogen activator (uPA), leading to excessive fibrinolysis and an increased bleeding tendency [10,11]. While the clinical phenotype of PAI-1 deficiency predominantly includes mucosal bleeding, menorrhagia, and postoperative hemorrhagic complications, spontaneous intracranial hemorrhage remains a rare presentation [12]. Due to the scarcity of reported cases, the natural history, therapeutic strategies, and long-term neurological outcomes of PAI-1 deficiency-related ICH in neonates remain largely unexplored.

## Case presentation

A 28-day-old female neonate was brought to a private hospital with complaints of irritability and altered sensorium. She was born to a second gravida mother at term gestation, with a birth weight of 3.5 kg. The baby was delivered vaginally, with an APGAR score of 8 and 9 at 1 and 5 minutes, respectively. The delivery was booked and supervised, with an uneventful antenatal and birth history. The other child in the family, a 5-year-old male, is healthy. Her birth history was unremarkable, and she had experienced an uneventful neonatal period. She was brought to the hospital with complaints of excessive crying, irritability, and refusal to feed. An initial clinical evaluation was performed, following which a cranial ultrasound (USG brain) was conducted. The USG revealed ventricular dilatation and the presence of intraventricular hemorrhage. Given these findings, an urgent non-contrast computed tomography (NCCT) of the head was performed, which demonstrated significant intraventricular and intracerebral hemorrhage, along with a midline shift.

On clinical examination, the neonate exhibited a pin-point right pupil and a bulging anterior fontanelle, suggestive of raised intracranial pressure. The overall clinical picture was indicative of ICH with impending neurological compromise, warranting urgent neurosurgical and critical care intervention. The baby was promptly stabilized with mechanical ventilation and supportive care due to her compromised neurological status. Laboratory evaluation revealed severe anemia (Hb: 5.9 g/dL), which was managed with packed red blood cell transfusion. Other lab parameters were in the normal range. Additionally, the neonate developed active seizures six hours after admission, which were effectively controlled with the administration of two intravenous anticonvulsant medications. To alleviate raised intracranial pressure, an EVD was placed with the help of a neurosurgery team. Despite these interventions, the neonate remained in critical condition. After 48 hours of intensive care at the initial hospital, she was transferred to another tertiary care center with ongoing mechanical ventilation and EVD in situ for further management, as per the family's decision based on their circumstances.

Initial stabilization

At the time of admission, the neonate was hemodynamically unstable, with vital parameters as follows: heart rate of 174 beats per minute, respiratory rate of 70 breaths per minute, oxygen saturation of 85% on 100% FiO₂, feeble peripheral pulses, mottled skin, cold peripheries, a capillary refill time (CRT) of 4 seconds, and blood pressure below the 5th percentile for age. The perfusion index was 0.32. Initial evaluation revealed a right-sided pneumothorax, further exacerbating the neonate's critical condition. An immediate chest X-ray confirmed the diagnosis, and an intercostal drain (ICD) was promptly inserted. The baby required intensive supportive care, including inotropic support, mechanical ventilation, and parenteral nutrition during the NICU stay. The neonate showed gradual clinical improvement, with shock resolving, allowing for the tapering and discontinuation of inotropes by day three. The pneumothorax resolved, and the ICD was removed on day five. However, the baby developed refractory seizures, necessitating treatment with three intravenous anticonvulsant drugs along with a midazolam infusion, which was gradually tapered and discontinued by day seven. Respiratory support was gradually weaned, with the baby transitioning from invasive mechanical ventilation to non-invasive ventilation (NIV) on day 8, followed by heated humidified high-flow nasal cannula (HHHFNC) on day 12, and subsequently to room air by day 23. The EVD was removed on day 10. Enteral feeds were initiated on day 3 and advanced to full enteral feeding by day 8. Cerebrospinal fluid (CSF) analysis was suggestive of meningitis, for which the neonate received a 21-day course of appropriate antibiotic therapy.

Etiological work-up and management

A comprehensive coagulation workup was undertaken to determine the underlying etiology of the neonate’s intracranial hemorrhage. Initial hematological investigations, including platelet count, platelet function tests, prothrombin time (PT), and activated partial thromboplastin time (aPTT), were within normal limits. Additionally, factor assays for common clotting disorders did not reveal any abnormalities. The diagnostic workup, including a sepsis screen, blood and CSF culture, and echocardiography, yielded normal results. The absence of systemic infection, inflammatory markers, and structural cardiac anomalies ruled out the common predisposing factors such as sepsis-associated coagulopathy, central nervous system infections (CSF cell count suggested meningitis which was probably a complication of EVD), and cardioembolic events as potential contributors to the hemorrhagic presentation. To rule out structural vascular anomalies contributing to the hemorrhage, magnetic resonance angiography (MRA) and magnetic resonance venography (MRV) were performed to investigate potential vascular anomalies that could have contributed to the ICH. The MRA findings ruled out cerebral aneurysms, arteriovenous malformations (AVMs), and other structural vascular abnormalities, eliminating these as possible etiologies. Similarly, MRV did not reveal any evidence of cerebral sinus venous thrombosis (CSVT), which is a known cause of neonatal intracranial hemorrhage due to impaired venous drainage and subsequent hemorrhagic infarction. Given the absence of acquired or common hereditary coagulation defects, a genetic evaluation was pursued. Clinical exome sequencing identified a heterozygous variant of uncertain significance in two genes: VWF and SERPINE1, the VWF gene is associated with Von Willebrand disease (VWD) types 1 and 3; however, further analysis of Von Willebrand factor (VWF) antigen levels and ristocetin cofactor activity revealed normal results, effectively ruling out VWD. The SERPINE1 gene is implicated in PAI-1 deficiency, a rare coagulation disorder. Functional assays confirmed significantly reduced PAI-1 levels, establishing the diagnosis of PAI-1 deficiency as the likely underlying cause of the neonate’s hemorrhagic presentation.

Rehabilitation was initiated in the NICU itself, incorporating a multidisciplinary approach aimed at optimizing neurodevelopmental outcomes. The rehabilitation strategy included physiotherapy, early stimulation, developmentally supportive care, and occupational therapy, along with supplementation with organ-specific nutraceuticals to support overall recovery. A family-centered approach was emphasized, actively involving the parents in the rehabilitation process to enhance the neonate’s long-term developmental potential. The confirmed diagnosis of PAI-1 deficiency provided a clear etiological explanation for the hemorrhagic events and guided targeted therapeutic interventions. To mitigate the bleeding risk associated with PAI-1 deficiency, antifibrinolytic therapy with TXA (20 mg/kg/day in two divided doses) was initiated as part of the management plan.

**Figure 1:**
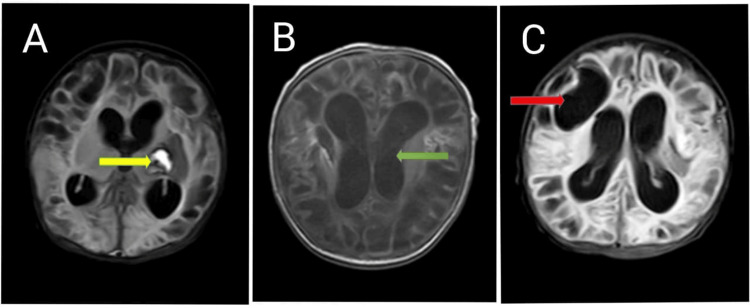
Pre discharge MRI revealed. (A) Hemorrhage in the lentiform nucleus (yellow arrow), (B) Dilatation of bilateral lateral ventricles (green arrow), (C) Cystic encephalomalacia (red arrow).

Pre-discharge magnetic resonance imaging (MRI) of the brain revealed cystic encephalomalacia, minimal residual intraventricular hemorrhage along with hemorrhage in the lentiform nucleus, and bilateral lateral ventricular dilatation, indicative of post-hemorrhagic changes and evolving cerebral injury (Figure 1).

At the time of discharge, the infant was hemodynamically stable and receiving TXA along with two oral anticonvulsant medications for seizure management. The baby was on exclusive breastfeeding with organ-specific nutraceutical supplementation. A structured follow-up plan was established for neurodevelopmental surveillance. On clinical examination, the infant demonstrated good sucking ability and overall activity. However, a neurological assessment using the Hammersmith infant neurological examination (HINE) yielded a total score of 43, indicative of an abnormal neurological outcome. Domain-specific sub-scores were as follows: cranial nerves (10/15), posture (10/18), movements (3/6), tone (13/24), and reflexes (8/15). The predominant deficits were observed in postural control, active movement initiation, and overall muscle tone regulation, consistent with the expected sequelae of neonatal hemorrhagic stroke. Given the high risk of motor impairment and cerebral palsy, the infant was discharged with recommendations for early and intensive neurorehabilitation, including regular physiotherapy and occupational therapy. Close monitoring was advised through a high-risk neonatal follow-up clinic, with additional follow-up in an early intervention center and regular assessment by a pediatric neurologist. Emphasis was placed on seizure control and the early detection of secondary complications, such as spasticity and sensorimotor deficits, to ensure timely interventions.

Parental and genetic counseling

Comprehensive parental counseling was provided regarding the child's neurological prognosis, potential motor impairment, and the importance of early intervention to optimize developmental outcomes. Parents were educated on home-based supportive care, early stimulation techniques, and the significance of adherence to physiotherapy and follow-up visits. Additionally, they were guided on recognizing early signs of developmental delay and seizure recurrence. Given the genetic diagnosis of PAI-1 deficiency, genetic counseling was conducted to explain the inheritance pattern, recurrence risk, and potential implications for future pregnancies. The family was informed about the availability of genetic testing for at-risk family members and the role of prenatal or preimplantation genetic screening in subsequent pregnancies if desired.

Follow-up

**Figure 2:**
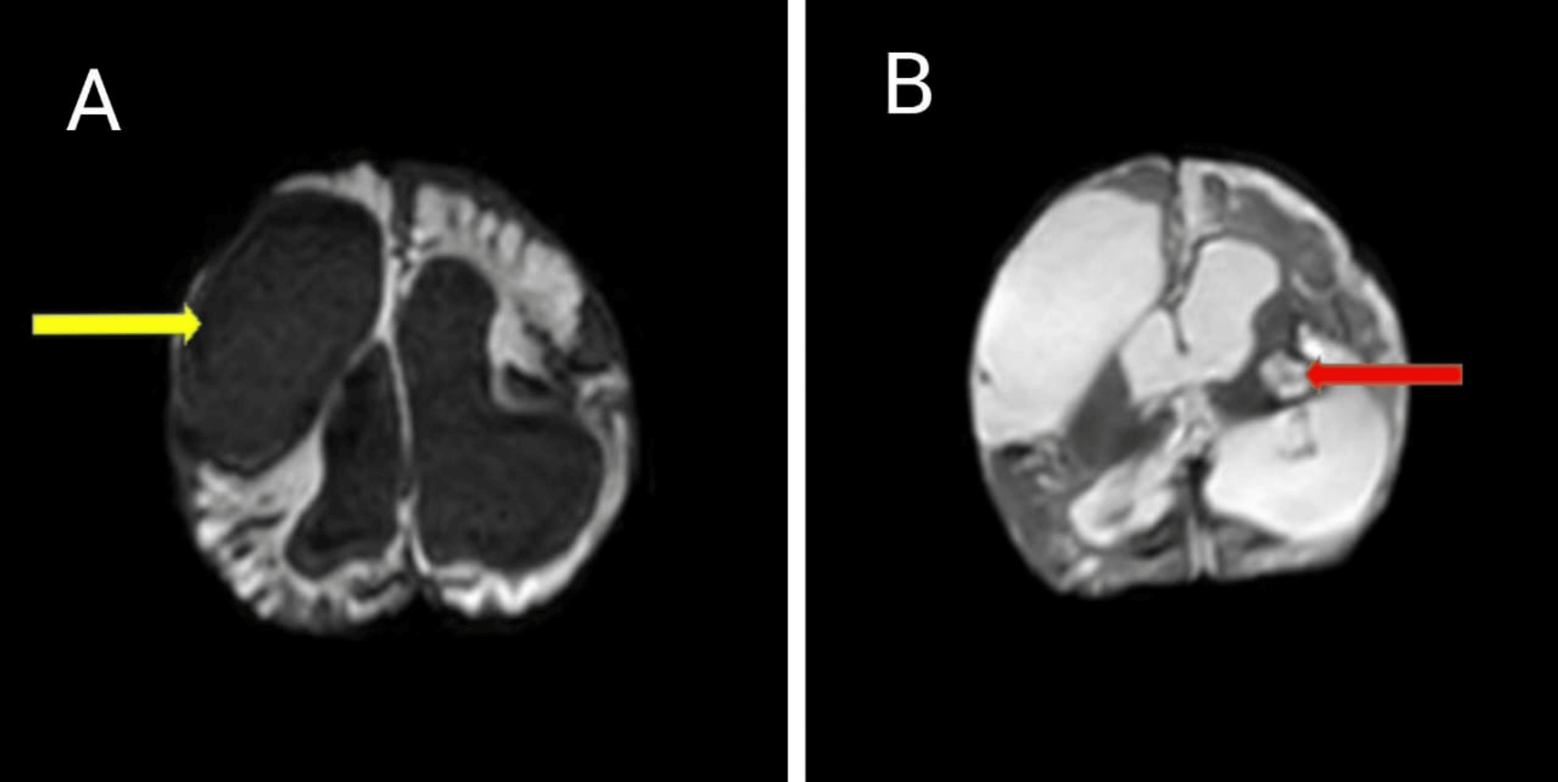
Follow up MRI at 6 months of age revealed. (A) cystic encephalomalacia (yellow arrow), (B) Chronic intraparenchymal hematoma in left thalamo capsular location with intraventricular extension into 3rd Ventricle (red arrow).

At the six-month follow-up, the infant had transitioned to a diet of breastfeeding supplemented with complementary feeds. The neurological assessment showed marginal improvement, as reflected by a HINE score of 49, although impairments persisted across all domains. Developmentally, the infant exhibited partial neck control and a social smile; however, gross motor, fine motor, language, and social milestones were significantly delayed, consistent with global developmental delay. Sensory evaluations revealed normal auditory function, but visual assessment raised concerns, warranting further ophthalmological evaluation and neurovisual rehabilitation. The persistence of motor deficits and tone abnormalities highlights the need for ongoing intensive neurodevelopmental therapy, with an expanded rehabilitation plan incorporating targeted visual stimulation exercises to optimize sensory-motor integration and functional development. The follow-up MRI at six months of age revealed cystic encephalomalacia and a chronic intraparenchymal hematoma localized to the left thalamo-capsular region, with intraventricular extension into the third ventricle. These findings are indicative of residual sequelae of prior hemorrhagic insult, contributing to the neurological deficits (Figure 2).

## Discussion

Perinatal ICH is a rare but potentially devastating condition, with perinatal stroke being one of its significant manifestations [13]. This case is particularly notable due to the identification of PAI-1 deficiency as the underlying etiology, a disorder rarely reported in the neonatal period. The findings emphasize the need for a comprehensive hematologic, genetic, and neurodevelopmental assessment in neonates presenting with unexplained ICH, particularly when conventional risk factors such as prematurity, birth trauma, sepsis, or structural vascular anomalies are absent. Although CSF analysis suggested meningitis, the blood and CSF cultures were sterile.

Establishing the diagnosis of PAI-1 deficiency in neonates presents a considerable challenge due to its rarity and lack of standardized screening tests. In this case, routine coagulation studies, including platelet function tests, prothrombin time (PT), activated partial thromboplastin time (aPTT), and coagulation factor assays, were normal, prompting the need for advanced molecular and functional assays.

Genetic sequencing identified a heterozygous variant in the* SERPINE1* gene, confirming PAI-1 deficiency as the underlying cause. Given the rarity of PAI-1 deficiency, standardized neonatal treatment protocols remain undefined. However, antifibrinolytic therapy with TXA has been proposed as a potential therapeutic intervention to prevent further hemorrhagic episodes [10,14-16]. In this case, TXA was administered as part of the management plan, along with multidisciplinary neurorehabilitation strategies. Given the absence of established guidelines for the optimal duration and discontinuation of TXA in perinatal stroke associated with PAI-1 deficiency, we propose a genotype-guided approach to therapy. Sanger sequencing will be performed to identify potential pathogenic variants in the PAI-1 gene. If the identified variant is classified as a variant of uncertain significance (VUS), TXA therapy will be discontinued due to the uncertain clinical impact. However, if the variant is determined to be pathogenic, TXA administration will continue to reduce the risk of recurrent hemorrhagic events. Despite aggressive management, the neonate developed neurological sequelae, including cystic encephalomalacia, post-hemorrhagic ventricular dilatation, and evolving motor deficits, evident from a low HINE score. These findings suggest a high risk of long-term neurodevelopmental impairment, necessitating early neurorehabilitation, structured follow-up, and continuous developmental surveillance [17,18].

Long-term management of this infant required comprehensive parental counseling, emphasizing the potential neurological sequelae, the importance of early intervention, and strategies to optimize neurodevelopmental outcomes. Parents were educated on the risk of motor impairment, seizure recurrence, and developmental delay, based on the neurological examination and imaging findings. Early intervention therapies, including physiotherapy, occupational therapy, and neurodevelopmental stimulation, were recommended to support optimal functional recovery. Nutritional and supportive care strategies were emphasized to optimize neurodevelopment, along with seizure monitoring and prevention of secondary complications such as spasticity and sensorimotor deficits. Additionally, parents were provided with home-based stimulation strategies to promote early developmental progress and were encouraged to participate in high-risk neonatal follow-up clinics and specialized neurorehabilitation programs.

Since PAI-1 deficiency is a rare inherited disorder, genetic counseling was conducted to inform the family about the inheritance pattern, recurrence risk, and future options. The genetic findings were explained in detail, highlighting that the heterozygous variant in the *SERPINE1* gene suggested a potential autosomal recessive or compound heterozygous inheritance, warranting further genetic analysis of the parents. The family was counseled regarding the potential risk of recurrence in subsequent pregnancies and advised on prenatal and preimplantation genetic testing options. Given the possibility of asymptomatic carriers within the family, genetic screening of close relatives was discussed as a precautionary measure.

## Conclusions

This case report emphasizes the importance of considering PAI-1 deficiency as a rare but significant differential diagnosis in unexplained neonatal hemorrhagic stroke. It highlights the need for early genetic and coagulation workup in neonates with spontaneous ICH, especially when standard investigations are inconclusive. Antifibrinolytic therapy, such as TXA, may be a potential treatment option, although further research is required to define its safety and long-term efficacy in neonates. Additionally, comprehensive neurodevelopmental follow-up, including early rehabilitation, vision, and hearing assessment, is crucial to optimizing long-term outcomes. Parental and genetic counseling should be offered to guide future reproductive planning and assess the risk for subsequent pregnancies. Given the rarity of neonatal PAI-1 deficiency and the absence of established treatment guidelines, further research is essential to develop standardized diagnostic, therapeutic, and follow-up protocols.
